# Clinical Outcomes Following Transition from ICS/LABA to Single-Inhaler Triple Therapy in Patients with Uncontrolled Asthma: A Real-World Cohort Study

**DOI:** 10.3390/medicina62081557

**Published:** 2026-08-14

**Authors:** Melis Yağdıran, Kurtuluş Aksu

**Affiliations:** Department of Allergy and Clinical Immunology, Ankara Atatürk Sanatorium Training and Research Hospital, University of Health Sciences, Ankara 06290, Türkiye; kurtulusaksu@yahoo.com

**Keywords:** asthma, single-inhaler triple therapy, ICS/LABA/LAMA, uncontrolled asthma, real-world study, exacerbation, asthma control, lung function

## Abstract

*Background and Objectives*: A considerable proportion of patients with asthma remain inadequately controlled despite treatment with inhaled corticosteroid/long-acting β_2_-agonist (ICS/LABA) therapy. Single-inhaler triple therapy comprising an inhaled corticosteroid, a long-acting β_2_-agonist, and a long-acting muscarinic antagonist (ICS/LABA/LAMA) has emerged as an effective step-up treatment; however, real-world evidence remains limited. This study aimed to evaluate the effects of transitioning from high-dose ICS/LABA therapy to single-inhaler triple therapy on asthma control, lung function, and exacerbation-related outcomes in routine clinical practice. *Materials and Methods*: This retrospective observational study included adult patients with uncontrolled asthma who were switched from high-dose ICS/LABA therapy to single-inhaler triple therapy at a tertiary referral center. Demographic characteristics, Asthma Control Test (ACT) scores, pulmonary function tests, blood eosinophil counts, exacerbation frequency, oral corticosteroid (OCS)-requiring exacerbations, short-acting β_2_-agonist (SABA) use, emergency department visits, and hospitalizations were compared before and after treatment escalation using paired statistical analyses. *Results*: Nineteen patients (mean age 58.9 ± 10.4 years; 63.2% female) were included. Following transition to single-inhaler triple therapy, asthma control improved significantly, with mean ACT scores increasing from 14.8 ± 3.2 to 23.4 ± 3.6 (*p* < 0.001). Mean forced expiratory volume in one second (FEV_1)_ increased from 1692.6 ± 722.7 mL to 2020.5 ± 871.5 mL, while predicted FEV_1_ improved from 64.2 ± 21.2% to 75.6 ± 21.6% (both *p* < 0.001). Total annual exacerbation frequency decreased from a median of 2 (interquartile range [IQR], 1–3) to 0 (IQR, 0–1; *p* = 0.0005), and 78.9% of patients experienced at least one fewer exacerbation. Significant reductions were also observed in OCS-requiring exacerbations, SABA use, emergency department visits, hospitalizations, and blood eosinophil counts. *Conclusions*: In this real-world cohort, transitioning from ICS/LABA therapy to single-inhaler triple therapy was associated with statistically significant improvements in asthma control and lung function, together with substantial reductions in exacerbation burden and healthcare utilization. These findings support the effectiveness of single-inhaler triple therapy as a step-up treatment strategy for patients with uncontrolled asthma in routine clinical practice.

## 1. Introduction

Asthma is a heterogeneous chronic airway disease affecting individuals of all ages and remains a major cause of morbidity worldwide [1]. Despite advances in treatment, many patients continue to experience poor symptom control, recurrent exacerbations, and impaired quality of life. These burdens are particularly pronounced among patients with moderate-to-severe asthma (Global Initiative for Asthma [GINA] steps 4–5), in whom exacerbations contribute substantially to healthcare utilization and disease-related morbidity [2,3]. Severe asthma phenotypes are characterized by persistent symptoms, frequent exacerbations, and ongoing treatment requirements despite optimized therapy [4]. Current international guidelines recommend inhaled corticosteroid (ICS) and long-acting β_2_-agonist (LABA) combination therapy as the cornerstone controller treatment for these patients [1,5].

However, a considerable proportion of patients remain inadequately controlled despite optimized ICS/LABA therapy [2,3]. For patients who continue to experience symptoms or exacerbations, the addition of a long-acting muscarinic antagonist (LAMA) is recommended as a step-up treatment option [1,6,7]. The benefits of adding LAMA to ICS/LABA therapy, including improvements in asthma control and reductions in exacerbation risk, have been consistently demonstrated in randomized controlled trials, systematic reviews, and meta-analyses [8,9].

Single-inhaler triple therapy (SITT), combining ICS, LABA, and LAMA within a single device, provides both anti-inflammatory and dual bronchodilator effects. By simultaneously targeting airway inflammation and bronchoconstriction, this therapeutic approach aligns with the treatable traits concept increasingly adopted in contemporary asthma management [10]. Pharmacological studies have demonstrated complementary bronchodilation between LABA and LAMA through complementary mechanisms acting on airway smooth muscle tone [11]. In large phase III clinical trials, single-inhaler triple therapy has been associated with significant improvements in lung function and reductions in asthma exacerbations compared with dual therapy regimens [6,7]. Furthermore, network meta-analyses have suggested that triple therapy may offer superior outcomes in terms of lung function and exacerbation prevention compared with ICS/LABA combinations alone [8,12].

Beyond its pharmacological advantages, delivering all three agents through a single inhaler may simplify treatment regimens, improve adherence, and reduce inhaler technique errors [13,14]. This is particularly important given the well-established association between poor treatment adherence and an increased risk of severe asthma exacerbations [15,16].

Despite the growing evidence supporting single-inhaler triple therapy, data from real-world clinical practice remain relatively limited. Real-life studies are essential because patient populations encountered in routine care often differ substantially from those enrolled in randomized controlled trials. In addition, single-inhaler triple therapy has only recently become reimbursed and more widely available in Türkiye, highlighting the need for national real-world evidence regarding its effectiveness in routine clinical practice.

Therefore, the present study aimed to evaluate the impact of transitioning from ICS/LABA therapy to single-inhaler triple therapy on asthma control, lung function, and exacerbation burden in a real-world cohort of patients with asthma.

## 2. Material and Methods

### 2.1. Research Method and Ethics Committee Approval

This retrospective descriptive clinical observational study was conducted at the Immunology and Allergy Diseases Clinic of our tertiary chest diseases hospital between 1 November 2022 and 30 October 2024, utilizing real-world data. All procedures complied with good clinical practice and the principles of the Declaration of Helsinki. Ethics approval was obtained from the Ankara Atatürk Sanatoryum Training and Research Hospital Clinical Research Ethics Committee (2024-BÇEK/415, 27 November 2025). No new interventions were applied; only existing clinical records were analyzed.

### 2.2. Patient Population

The study included patients with moderate to severe asthma (GINA steps 4–5) who were followed in our clinic during the specified period. Treatment escalation was performed according to GINA recommendations and routine clinical practice. Patients who remained uncontrolled despite ICS/LABA treatment were individually evaluated, and the choice of further treatment (including single-inhaler triple therapy or biologic therapy) was based on the patient’s clinical characteristics, type 2 inflammatory profile, eligibility for biologic therapy, reimbursement criteria, and patient preference. Eligible patients were those receiving high-dose inhaled corticosteroid (ICS) and long-acting β_2_-agonist (LABA) combination therapy who remained uncontrolled, defined as an Asthma Control Test (ACT) score < 20 at the time of treatment escalation. All patients were switched from high-dose ICS/LABA to high-dose single-inhaler triple therapy with fluticasone furoate/umeclidinium/vilanterol (Trelegy Ellipta, GlaxoSmithKline, Brentford, UK; 200/62.5/25 μg).

To ensure adequate comparison, only patients with at least 12 months of follow-up data both before and after the initiation of triple therapy were included in the analysis.

Patients with incomplete or missing clinical records, irregular follow-up, a diagnosis of asthma–chronic obstructive pulmonary disease (COPD) overlap or other significant chronic pulmonary diseases, and those receiving biologic therapy during the study period were excluded. In addition, patients who had been treated with a long-acting muscarinic antagonist (LAMA) via a separate inhaler device in addition to ICS/LABA prior to initiation of single-inhaler triple therapy were not eligible for inclusion. Demographic characteristics, comorbidities, and treatment histories were reviewed for all eligible patients.

### 2.3. Data Acquisition

Patient data were retrospectively collected from medical records and the hospital information management system. Collected variables included demographics (age, sex, body mass index, smoking status), comorbidities, concomitant medications, asthma diagnosis date, ICS/LABA and triple therapy initiation dates, and treatment adherence as documented in patient files.

Laboratory parameters (blood eosinophil counts), pulmonary function tests (forced expiratory volume in one second [FEV_1_], forced vital capacity [FVC], FEV_1_/FVC ratio, and peak expiratory flow [PEF]), Asthma Control Test (ACT) scores, annual asthma exacerbation frequencies, emergency department visits, hospitalizations, and short-acting beta-agonist (SABA) usage were recorded for both treatment periods.

Spirometric measurements were performed according to the 2019 American Thoracic Society/European Respiratory Society (ATS/ERS) standards prior to bronchodilator administration.

Data obtained from the 12-month period before and the 12-month period after initiation of single-inhaler triple therapy were compared.

### 2.4. Definitions

Treatment adherence was assessed retrospectively based on physician documentation in the medical records during routine follow-up visits. Patients were considered adherent if regular inhaler use was documented in the clinical notes. No validated adherence questionnaire or objective adherence measure was available because of the retrospective study design.

Asthma exacerbations were identified from physician-documented episodes of worsening asthma in the medical records. Total annual exacerbation frequency represented the total number of documented exacerbation episodes during each 12-month observation period, irrespective of whether they required OCS treatment, an emergency department visit, or hospitalization. OCS-requiring exacerbations, asthma-related emergency department visits, and hospitalizations were also recorded and analyzed separately as specific exacerbation-related outcomes. Severe exacerbations were defined according to the ATS/ERS recommendations as worsening of asthma requiring systemic corticosteroid treatment for at least 3 days, an increase in the maintenance systemic corticosteroid dose for at least 3 days, or an asthma-related emergency department visit or hospitalization [17].

Asthma control was evaluated using the Asthma Control Test (ACT), a validated five-item questionnaire assessing symptoms over the previous four weeks. ACT scores range from 5 to 25, with scores ≥ 20 indicating well-controlled asthma and scores < 20 indicating uncontrolled disease.

### 2.5. Statistical Analysis

Statistical analyses were performed using SPSS version 26 (IBM Corp., Armonk, NY, USA).

Normality of data distribution was assessed using the Shapiro–Wilk test. Continuous variables were expressed as mean ± standard deviation (SD) or median (interquartile range, IQR) as appropriate. Categorical variables were presented as frequencies and percentages.

Comparative analyses between the ICS/LABA and ICS/LABA/LAMA treatment periods were performed using paired-sample *t*-tests or Wilcoxon signed-rank tests according to data distribution.

A *p* value < 0.05 was considered statistically significant.

Due to the retrospective design of the study, no a priori sample size calculation was performed.

## 3. Results

A total of 19 patients with asthma receiving high-dose ICS/LABA combination therapy were included in the study. Twelve patients (63.2%) were female and seven (36.8%) were male. The mean age was 58.9 ± 10.4 years (range, 42–79 years), and the mean body mass index (BMI) was 29.6 ± 4.1 kg/m^2^. Atopy was present in 10 patients (52.6%), and three patients (15.8%) were current smokers. Treatment adherence was high during both the ICS/LABA period and the subsequent single-inhaler triple therapy period in all patients. Baseline demographic and clinical characteristics are summarized in Table 1.

The mean baseline eosinophil count during the ICS/LABA treatment period was 435.0 ± 260.0 cells/µL (median, 380; IQR, 305–550). A significant reduction in eosinophil levels was observed following initiation of single-inhaler triple therapy (295.3 ± 226.7 cells/µL; *p* = 0.014).

**Figure 1:**
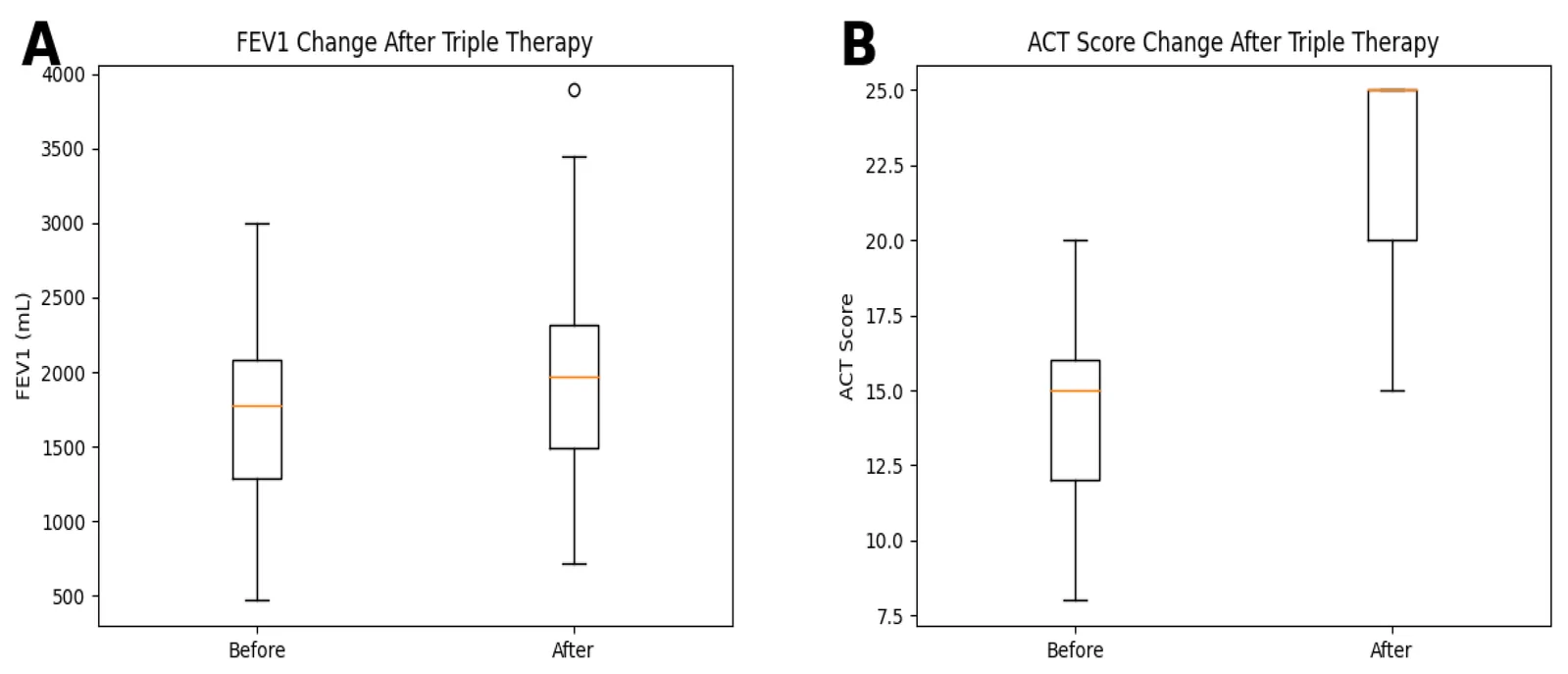
(**A**) Change in forced expiratory volume in one second (FEV_1_) following triple therapy. (**B**) Increase in Asthma Control Test (ACT) scores after treatment, indicating improved symptom control.

Lung function improved significantly after switching to single-inhaler triple therapy. Mean FEV_1_ (% predicted) increased from 64.2 ± 21.2% to 75.6 ± 21.6% (*p* < 0.001), corresponding to an absolute improvement of approximately 11 percentage points. Similarly, absolute FEV_1_ values (mL) increased significantly from 1692.6 ± 722.7 mL to 2020.5 ± 871.5 mL (*p* < 0.001). FVC values also demonstrated an increasing trend following triple therapy. However, no statistically significant change was observed in the FEV_1_/FVC ratio (*p* > 0.05). Changes in lung function and ACT scores are illustrated in Figure 1.

Transition from ICS/LABA therapy to single-inhaler triple therapy was associated with marked and statistically significant improvements in clinical outcomes (Table 2).

**Figure 2:**
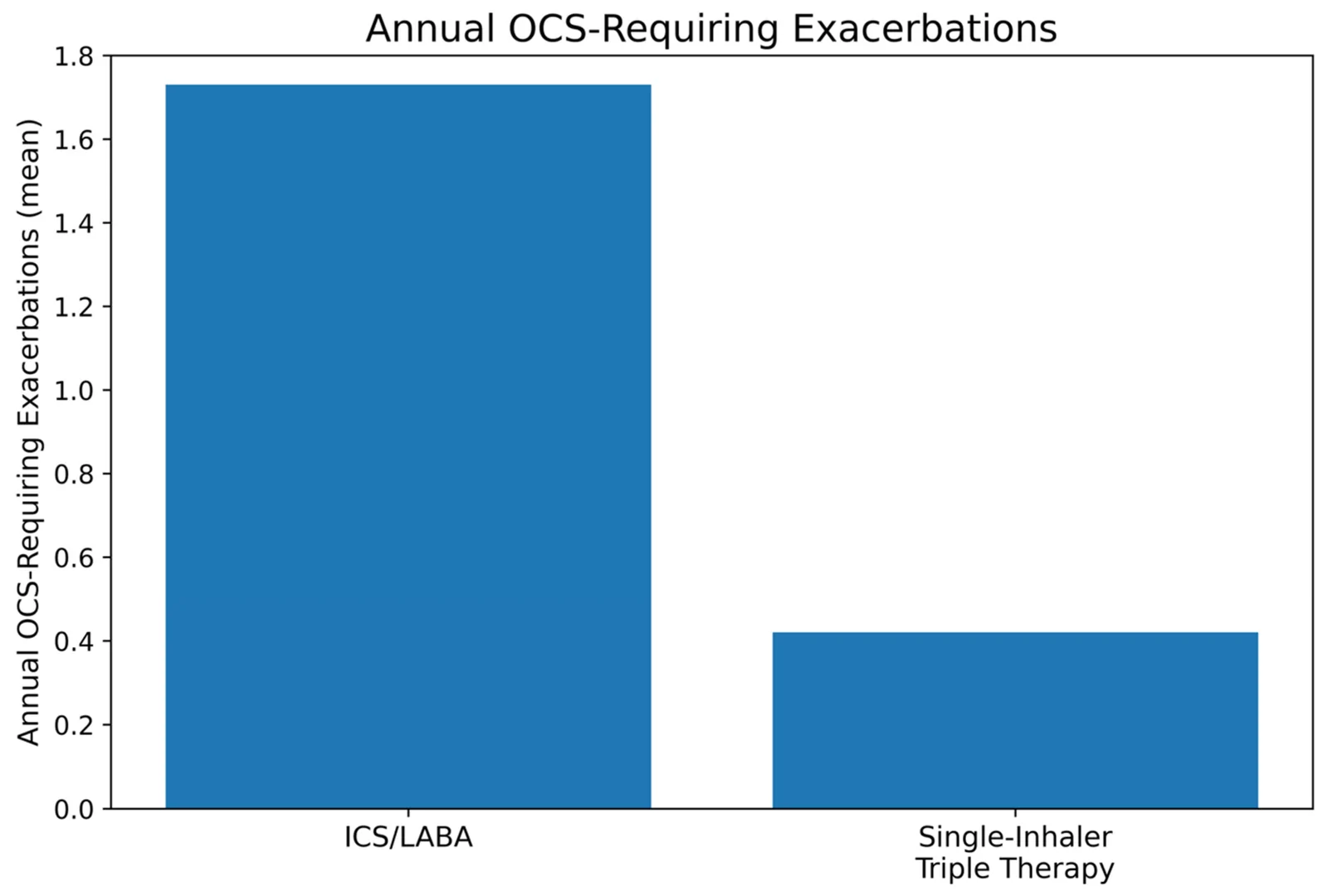
Reduction in mean annual oral corticosteroid (OCS)-requiring exacerbations after initiation of single-inhaler triple therapy.

The mean Asthma Control Test (ACT) score increased from 14.8 ± 3.2 to 23.4 ± 3.6 (*p* < 0.001). The proportion of patients achieving well-controlled asthma (ACT ≥20) increased substantially following treatment escalation. Annual exacerbation frequency decreased significantly (Wilcoxon signed-rank test, *p* = 0.0005), with the median number of exacerbations declining from 2 (IQR, 1–3) to 0 (IQR, 0–1) per year. A reduction of at least one annual exacerbation was observed in 15 patients (78.9%). The annual number of oral corticosteroid (OCS)-requiring exacerbations decreased significantly from 1.73 ± 1.09 to 0.42 ± 0.50 (*p* < 0.001), as shown in Figure 2.

The annual number of oral corticosteroid (OCS)-requiring exacerbations decreased significantly from 1.73 ± 1.09 to 0.42 ± 0.50 (*p* < 0.001). Emergency department visits were reduced from 0.68 ± 1.20 to 0.05 ± 0.23 per year (*p* = 0.035), while hospitalizations decreased from 0.42 ± 0.51 to 0.05 ± 0.23 per year (*p* = 0.004). Short-acting β_2_-agonist (SABA) use also declined significantly during the triple therapy period (*p* < 0.001).

## 4. Discussion

In this real-world cohort of patients with uncontrolled asthma receiving high-dose ICS/LABA therapy, transitioning to single-inhaler triple therapy was associated with significant improvements in asthma control, lung function, and exacerbation outcomes. We observed substantial increases in ACT scores and FEV_1_ values, accompanied by marked reductions in annual exacerbation frequency, oral corticosteroid use, emergency department visits, and hospitalizations. Notably, nearly four out of five patients experienced at least one fewer exacerbation during follow-up, highlighting the effectiveness of triple therapy in routine clinical practice [1,2].

Current asthma guidelines recommend the addition of a LAMA in patients who remain symptomatic despite optimized ICS/LABA treatment [1]. This recommendation is supported by randomized controlled trials demonstrating that tiotropium add-on therapy improves lung function and reduces exacerbation risk [9,18]. More recently, comprehensive reviews and expert consensus documents have further emphasized the role of single-inhaler triple therapy as an effective step-up strategy [10,19,20].

The low baseline ACT scores observed in our cohort indicate that asthma control was insufficient despite ongoing ICS/LABA treatment. Similar observations have been reported in real-world cohorts reporting substantial rates of uncontrolled asthma among patients receiving guideline-based controller therapy [2,3,14,15]. Following initiation of triple therapy, ACT scores increased by approximately 8–9 points, well above the established minimal clinically important difference for the ACT [21]. These results suggest that the addition of a LAMA and the resulting enhancement of bronchodilation can translate into statistically significant improvements in patient-reported outcomes [5,10,11,12,13]. However, because all patients were enrolled during a period of poor asthma control (ACT < 20), part of the observed improvement in ACT scores may reflect regression to the mean rather than the treatment effect alone. Therefore, the magnitude of the observed improvement should be interpreted with caution in light of the uncontrolled study design.

Frequent SABA use is increasingly recognized as a marker of poor asthma control and a predictor of future exacerbations [1,22]. Therefore, the significant reduction in SABA use observed in our study likely reflects not only symptomatic improvement but also a broader improvement in overall disease control.

The efficacy of single-inhaler triple therapy has been established in several randomized clinical trials. In the CAPTAIN study, FF/UMEC/VI significantly improved lung function and reduced exacerbation risk compared with dual therapy [6]. Similar benefits were reported in the TRIMARAN and TRIGGER trials, which demonstrated improvements in both lung function and exacerbation outcomes with triple therapy [7]. These outcomes have subsequently been reinforced by systematic reviews and expert consensus documents supporting the use of single-inhaler triple therapy in patients with uncontrolled asthma [10,19,20]. The magnitude and direction of the treatment effects observed in our cohort were comparable with those reported in these trials.

In recent years, emerging real-world evidence has further strengthened the role of single-inhaler triple therapy in asthma management. In the REAL ASTHMA study, Kushima and colleagues reported significant improvements in asthma control and lung function after switching from ICS/LABA therapy to FF/UMEC/VI in symptomatic patients with asthma [23]. Likewise, a recent systematic review of real-world effectiveness studies found consistent reductions in exacerbations and improvements in disease control following initiation of ICS/LAMA/LABA therapy [24]. Furthermore, Baptist et al. demonstrated favorable clinical outcomes after initiation of FF/UMEC/VI in a Medicare population previously treated with ICS/LABA [25]. The consistency between these studies and our results suggests that the benefits of single-inhaler triple therapy are reproducible across different healthcare settings and patient populations.

We observed significant improvements in FEV_1_ following initiation of triple therapy. Although the FEV_1_/FVC ratio did not change significantly, this does not necessarily indicate a lack of physiological benefit. According to the ATS/ERS spirometry standards, FEV_1_/FVC primarily reflects the presence of airflow obstruction, whereas FEV_1_ is more sensitive to changes in airway caliber [26]. Similar improvements in FEV_1_ without substantial changes in the FEV_1_/FVC ratio have also been reported in major randomized clinical trials evaluating single-inhaler triple therapy [6,7]. Therefore, the observed increase in FEV_1_ may reflect meaningful improvement in airway function despite persistence of an obstructive pattern.

Blood eosinophil counts decreased significantly during the triple therapy period. This may partly be explained by the anti-inflammatory effects of the ICS component on systemic type 2 inflammation. However, we did not identify an independent relationship between eosinophil levels and clinical response. Although previous studies have shown that blood eosinophil counts of ≥150 or ≥300 cells/µL are associated with exacerbation risk and may predict treatment response [27,28], our findings suggest that the benefits of triple therapy may not be restricted to a specific inflammatory phenotype. Similar conclusions were drawn from recent analyses from the CAPTAIN program, which showed that treatment response may vary according to baseline inflammatory characteristics, yet clinically relevant improvements can still be observed across a broad range of patient phenotypes [29,30]. The observed reduction in blood eosinophil counts is unlikely to be attributable to the addition of LAMA itself, as LAMA has no established direct effect on eosinophilic inflammation. All patients in our cohort were receiving high-dose ICS/LABA before switching to high-dose single-inhaler triple therapy (FF/UMEC/VI 200/62.5/25 μg); therefore, the inhaled corticosteroid dose remained broadly comparable after the treatment switch. Consequently, the observed reduction in blood eosinophil counts is unlikely to be explained solely by increased ICS exposure. Improved adherence associated with the simplified single-inhaler regimen may partly explain this finding, although this remains speculative because adherence was not objectively assessed.

The mechanisms underlying the benefits of triple therapy likely extend beyond suppression of type 2 inflammation alone. Contemporary asthma management increasingly emphasizes the concept of treatable traits, in which modifiable characteristics such as airway inflammation, bronchoconstriction, and inhaler-related factors are targeted individually [10]. Within this framework, triple therapy offers a complementary therapeutic approach by combining anti-inflammatory activity with dual bronchodilation [19,20,31]. The complementary effects of LABA and LAMA on airway smooth muscle tone may contribute substantially to the clinical improvements observed in our cohort [11,19,31].

Another important advantage of single-inhaler triple therapy is the potential to improve treatment adherence. Incorrect inhaler technique and poor adherence remain major barriers to achieving asthma control [14,15]. In a large real-world study, Busse et al. demonstrated higher adherence and treatment persistence among patients receiving single-inhaler compared with multiple-inhaler triple therapy [32]. Similar treatment effects were reported in elderly patients, in whom single-inhaler therapy was associated with improved treatment continuity and medication persistence [33]. More recently, Oga et al. also confirmed favorable adherence to ICS/LAMA/LABA therapy in routine clinical practice [34]. Although adherence was not formally measured in our study, all patients were considered highly adherent during follow-up, and the favorable outcomes observed may partly reflect the simplified treatment regimen offered by a single-inhaler approach.

The magnitude of the observed improvements in asthma control, lung function, and exacerbation burden further supports the clinical relevance of our findings. These results indicate that the benefits of triple therapy were not only statistically significant but also meaningful from a patient-centered perspective.

The strengths of our study include its real-world design, paired within-patient analysis, and the simultaneous evaluation of clinical and functional outcomes. By specifically examining patients who transitioned from ICS/LABA therapy to single-inhaler triple therapy, we were able to assess treatment-related changes at the individual patient level. Given the recent reimbursement and increasing availability of single-inhaler triple therapy in Türkiye, real-world data in this specific population remain limited [19,20,24,25,31]. Our findings therefore provide contemporary national evidence that may complement existing clinical trial data. Our findings are also consistent with the systematic review and meta-analysis by Kim et al., which demonstrated that single-inhaler triple therapy was associated with improved asthma control and a reduced risk of exacerbations compared with dual therapy across randomized clinical trials [35].

Beyond the statistical significance of these outcomes, the marked increase in ACT scores and reduction in OCS-requiring exacerbations may be particularly meaningful from the patient perspective. In a discrete choice experiment involving patients with severe asthma, avoidance of OCS and improvement in asthma control measured by ACT were prioritized over lung function and exacerbation-related attributes when defining remission. Although patient preferences and remission were not directly assessed in our study, these findings suggest that the improvements observed in ACT and OCS-related outcomes may represent benefits that are highly valued by patients and may enhance treatment satisfaction and persistence [36].

Several limitations should also be acknowledged. The retrospective single-center design, small sample size, and absence of a control group may limit the generalizability of the results. Furthermore, excluding patients with incomplete clinical records or irregular follow-up may have introduced selection bias. In addition, the lack of a control group makes it difficult to distinguish the observed treatment effects from the natural variation in disease course over time. Nevertheless, the paired analysis design allowed each patient to serve as their own control, thereby reducing the impact of interindividual variability. The relatively small sample size may also have limited the statistical power to detect significant differences in some outcomes, such as the FEV_1_/FVC ratio. Therefore, non-significant findings should be interpreted with caution and should not necessarily be considered evidence of the absence of a treatment effect. Larger prospective multicenter studies are needed to confirm these findings.

Furthermore, the present study was not designed to assess clinical remission, and the more stringent target of sustained absence of exacerbations together with high asthma control and stable or normalized lung function could not be evaluated. Because no universally accepted definition of asthma remission currently exists, our findings should not be interpreted as evidence that remission was achieved. Biologic therapies may result in clinical remission in a proportion of appropriately selected patients with severe asthma [37]. Although treatment escalation was consistent with GINA recommendations, the final selection of single-inhaler triple therapy was individualized and influenced by patient preference, clinical characteristics, biologic eligibility, reimbursement criteria, and physician judgment rather than a prespecified biomarker-driven treatment algorithm. More comprehensive phenotyping using blood eosinophils, fractional exhaled nitric oxide, and serum IgE might have identified patients for whom biologic therapy could have represented a more effective and potentially cost-efficient strategy. Therefore, treatment-selection bias cannot be excluded, and the present study cannot establish the comparative effectiveness of single-inhaler triple therapy versus biologic treatment.

An additional potential source of bias is the expectation of reducing or avoiding OCS use. A recent discrete choice experiment showed that both patients and healthcare professionals were willing to trade some treatment benefits to avoid OCS-related adverse effects [38]. This expectation may have increased the initial acceptance of and adherence to single-inhaler triple therapy, thereby contributing to the observed improvements. Because adherence was assessed only from clinical documentation and follow-up was limited to 12 months, this potential expectancy-related effect could not be quantified. In patients who continued to experience exacerbations despite triple therapy, treatment satisfaction and persistence might decline over longer follow-up; prospective studies are required to evaluate this possibility.

In conclusion, among patients with uncontrolled asthma receiving ICS/LABA therapy, transition to single-inhaler triple therapy was associated with significant reductions in exacerbations, improvements in asthma control, and lung function. Our findings support and extend the results of randomized clinical trials by demonstrating the effectiveness of single-inhaler triple therapy in routine clinical practice.

## 5. Conclusions

Switching from ICS/LABA to single-inhaler triple therapy was associated with significant improvements in asthma control, lung function, exacerbation frequency, oral corticosteroid use, emergency department visits, and blood eosinophil counts over 12 months in patients with uncontrolled asthma. These findings support the effectiveness of single-inhaler triple therapy in routine clinical practice and suggest that it represents a valuable treatment option for patients who remain uncontrolled despite ICS/LABA therapy. Larger prospective studies are warranted to confirm these findings.

## Figures and Tables

**Table 1:** Baseline Characteristics of the Study Population (n = 19).

Variable	Value
Female, n (%)	12 (63.2)
Male, n (%)	7 (36.8)
Age, years, mean ± SD	58.9 ± 10.4
BMI, kg/m^2^, mean ± SD	29.6 ± 4.1
Atopy, n (%)	10 (52.6)
Current smokers, n (%)	3 (15.8)
Baseline ACT score, mean ± SD	14.8 ± 3.2
Baseline FEV_1_ (% predicted), mean ± SD	64.2 ± 21.2
Baseline FEV_1_ (mL), mean ± SD	1692.6 ± 722.7
Baseline FEV_1_/FVC (%), mean ± SD	76.1 ± 13.2
Total annual exacerbations, median (IQR)	2 (1–3)

Abbreviations: ACT, Asthma Control Test; BMI, body mass index; FEV_1_, forced expiratory volume in 1 s; FVC, forced vital capacity; IQR, interquartile range; SD, standard deviation.

**Table 2:** Comparison of Clinical Outcomes During ICS/LABA and Single-Inhaler Triple Therapy Periods (n = 19).

Parameter	ICS/LABA	Single-Inhaler Triple Therapy	*p* Value
ACT score, mean ± SD	14.8 ± 3.2	23.4 ± 3.6	<0.001
FEV_1_ (% predicted), mean ± SD	64.2 ± 21.2	75.6 ± 21.6	<0.001
FEV_1_ (mL), mean ± SD	1692.6 ± 722.7	2020.5 ± 871.5	<0.001
FEV_1_/FVC (%), mean ± SD	76.1 ± 13.2	73.7 ± 20.8	>0.05
Total annual exacerbations, median (IQR)	2 (1–3)	0 (0–1)	0.0005
OCS-requiring exacerbations, mean ± SD	1.73 ± 1.09	0.42 ± 0.50	<0.001
Emergency department visits, mean ± SD	0.68 ± 1.20	0.05 ± 0.23	0.035
Hospitalizations, mean ± SD	0.42 ± 0.51	0.05 ± 0.23	0.004
SABA use, mean ± SD	1.11 ± 1.59	0.11 ± 0.32	<0.001
Blood eosinophils (cells/µL), mean ± SD	435.0 ± 260.0	295.3 ± 226.7	0.014

Abbreviations: ACT, Asthma Control Test; FEV_1_, forced expiratory volume in 1 s; FVC, forced vital capacity; ICS, inhaled corticosteroid; IQR, interquartile range; LABA, long-acting β_2_-agonist; OCS, oral corticosteroid; SABA, short-acting β_2_-agonist; SD, standard deviation.

## Data Availability

The data presented in this study are available from the corresponding author upon reasonable request. The data are not publicly available because of privacy and ethical restrictions.
